# Use of Three-dimensional Mapping to Identify an Alternating Atrial Activation Pattern in the Coronary Sinus

**DOI:** 10.19102/icrm.2018.090504

**Published:** 2018-05-15

**Authors:** Sri A. Sundaram, Chas D. Boorman, Nate A. Mullins, J. Ryan Jordan, William C. Choe

**Affiliations:** ^1^South Denver Cardiology Associates, Littleton, CO, USA; ^2^Abbott Laboratories, Chicago, IL, USA

**Keywords:** Alternating, atypical flutter, coronary sinus, three-dimensional mapping

## Abstract

Atypical left atrial flutters present following atrial fibrillation ablation have been well-documented in the literature. These arrhythmias are known to be difficult to localize and ablate. An atypical flutter with an alternating activation pattern in the coronary sinus, however, is unusual and has rarely been discussed. In this case report, we describe the use of high-density three-dimensional anatomic mapping to successfully localize and terminate an atypical flutter with an alternating atrial activation pattern in the coronary sinus.

## Case presentation

A 66-year-old male with a history of drug-refractory, symptomatic persistent atrial fibrillation who had undergone an initial catheter-based ablation one year prior presented to the clinic. An antral ablation was performed with a contact-sensing, irrigated-tip radiofrequency catheter. Entrance and exit block were confirmed and the case was concluded. No additional ablation lines were placed.

**Figure 1:**
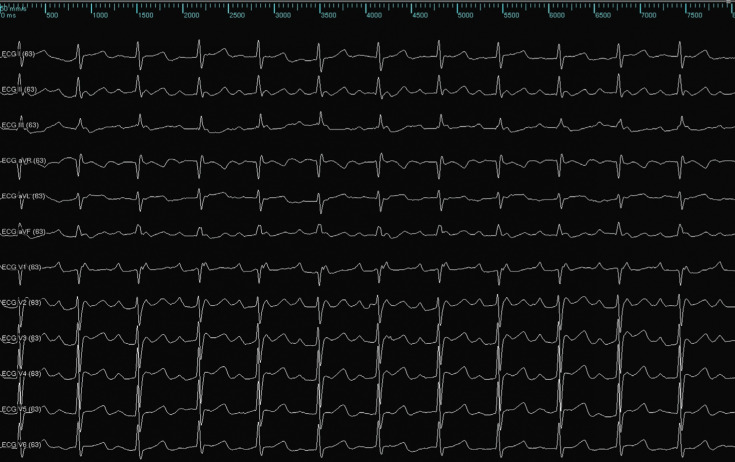
Surface electrocardiogram of the presenting tachycardia.

Following the three-month blanking period, the patient experienced several episodes of atypical atrial flutter that was refractory to antiarrhythmic medications. After further recurrent episodes, the patient chose to undergo a repeat ablation procedure **(Figure 1)**.

During the second ablation procedure, a decapolar coronary sinus (CS) catheter (LiveWire; Abbott Laboratories, Chicago, IL, USA) was advanced into the CS and shadowed to maintain a stable reference throughout the case. Two separate transseptal punctures were performed. A fixed sheath (Daig SL-1; Abbott Laboratories, Chicago, IL, USA) and a steerable transseptal sheath (Agilis; Abbott Laboratories, Chicago, IL, USA) were inserted into the left atrium (LA).

**Figure 2:**
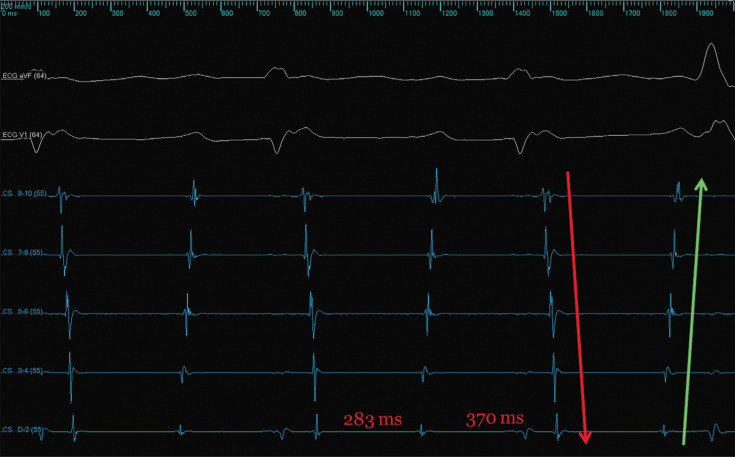
Alternating activation pattern in the CS with a proximal to distal pattern with a CL of 283 ms and a distal to proximal CS pattern with a CL of 370 ms. The green arrow highlights the proximal to distal CS pattern and the red arrow highlights the distal to proximal CS pattern. ECG: echocardiogram; CS: coronary sinus.

Using an impedance-based electroanatomic three-dimensional (3D) mapping system (EnSite™ Precision™; Abbott Laboratories, Chicago, IL, USA), geometry of the LA and the pulmonary veins (PVs) were acquired using a circular mapping catheter (Reflexion™ Spiral; Abbott Laboratories, Chicago, IL, USA). The initial CS electrograms showed two separate cycle lengths (CLs). Tachycardia no. 1 had a CL of 283 ms and a propagation pattern of proximal to distal on the CS catheter, while tachycardia no. 2 had a CL of 370 ms and demonstrated distal to proximal CS activation. These two tachycardias alternated every other beat, based on CS activation **(Figure 2)**.

High-density mapping of the LA was performed with high-density activation sequence mapping in combination with voltage gradient mapping overlay (HD-VGM). The configuration of the mapping system has been previously described in detail.^1,2^ Briefly, the EnSite™ Precision™ mapping system was set to display all of the mapping points with only eight isochronal color bands in the following activation pattern: white → red → orange → yellow → green → light blue → dark blue → purple. Each color band represents equally spaced timing. The activation pattern of the tachycardia can be followed by the color sequence. Mapping was performed in four minutes and 4,210 points were obtained and used to create a 3D anatomic map for both arrhythmias. The data for both arrhythmias were collected together but were subsequently analyzed individually. In addition, the map was set up initially using the CS 5–6 electrode as the mapping reference. When the alternating CL presented, the reference was changed to the distal CS, as this was the electrode with the most variation between the two CLs. With each map, 100% of the CL was obtained within the LA.

**Figure 3:**
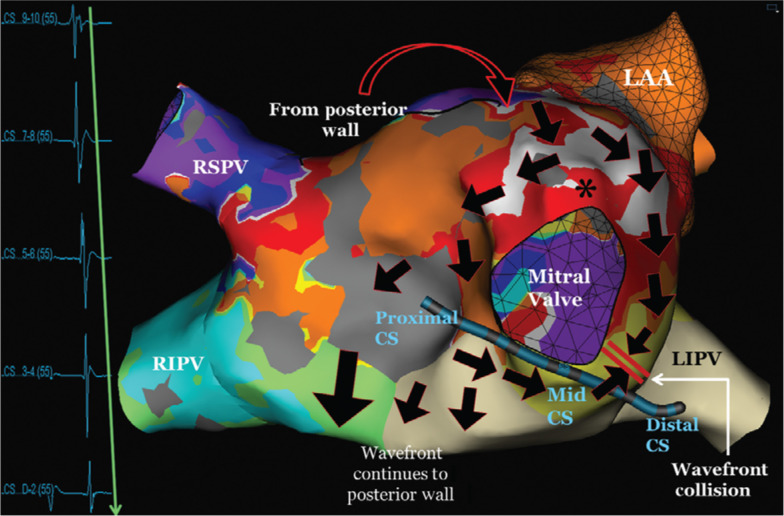
3D anatomic mapping of the proximal to distal activation in the CS. The earliest location of the tachycardia is noted by the white region that then spreads in both clockwise and counterclockwise patterns around the mitral valve. The counterclockwise activation accounts for the proximal to distal pattern in the CS catheter. Both wave fronts collide in the area marked by the double bars. The tachycardia also continues around the remainder of the LA, as noted by the large arrows. *Point at which the ERP of the tissue is reached and the tissue becomes refractory. 3D: three-dimensional; CS: coronary sinus; RSPV: right superior pulmonary vein; LAA: left atrial appendage; RIPV: right inferior pulmonary vein; LIPV: left inferior pulmonary vein.

3D anatomic mapping was used to identify both arrhythmias. The first tachyarrhythmia was at a cycle length of 283 ms. Mapping showed this to be a macroreentrant tachycardia with the earliest activation at approximately 12 o’clock on the mitral valve. The tachycardia had a counterclockwise arm around the mitral valve that activated the CS in a proximal to distal pattern **(Figure 3)**. In addition, there was a clockwise arm that travelled down the lateral side of the mitral valve. The counterclockwise and clockwise arms collided in the yellow area marked with double bars. The macroreentrant circuit then continued to the posterior wall, traveling superiorly back to the roof and then to the 12 o’clock mitral valve area.

**Figure 4:**
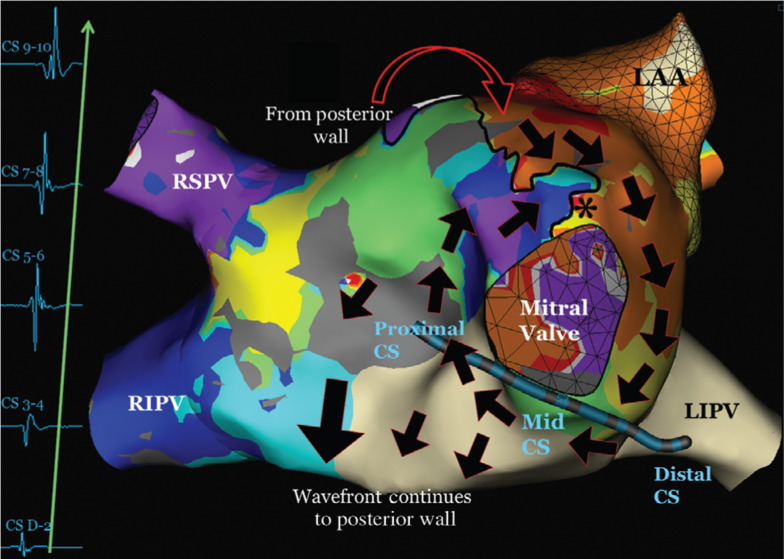
3D anatomic mapping of the distal to proximal activation pattern in the CS. The wave front travels only in a clockwise pattern around the mitral valve. The ERP of the tissue, marked by the asterisk, must have been reached, so it does not conduct. The dark black line indicates an area of functional block. The activation then follows a clockwise pattern around the mitral valve and leads to a distal to proximal CS pattern. The tachycardia also continues around the remainder of the LA, as noted by the large arrows. *Point at which the ERP of the tissue is reached and the tissue becomes refractory. 3D: three-dimensional; CS: coronary sinus; RSPV: right superior pulmonary vein; LAA: left atrial appendage; RIPV: right inferior pulmonary vein; LIPV: left inferior pulmonary vein.

The second tachyarrhythmia was also mapped and was also a macroreentrant arrhythmia, but with a cycle length of 370 ms. Our map displayed the earliest area in white. It then traveled from red → yellow → orange → green → light blue → purple. The arrhythmia traveled in a clockwise pattern around the mitral valve. This led to a distal to proximal pattern in the CS. The early meets late part of this tachycardia was at approximately the 12 o’clock position, just above the mitral valve. The circuit again continued to the posterior wall, traveling superiorly back to the 12 o’clock mitral valve area **(Figure 4)**.

## Discussion

**Figure 5:**
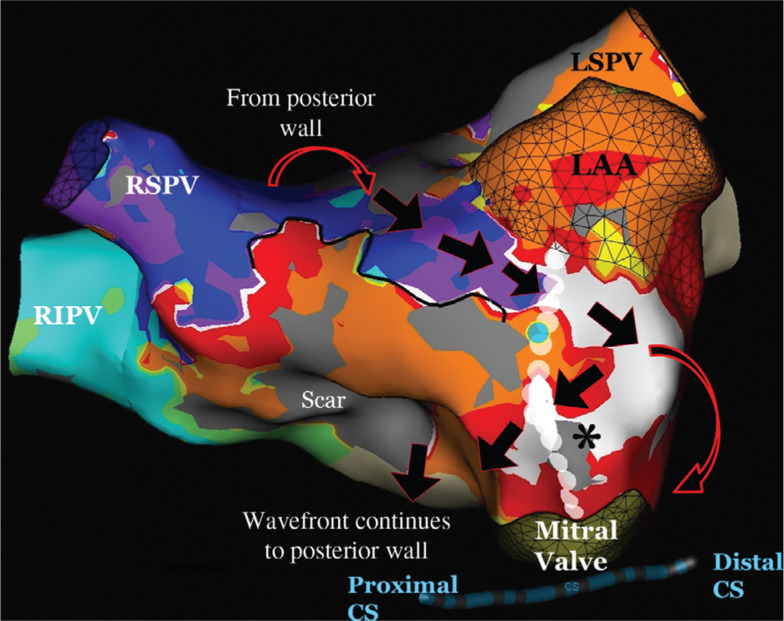
3D anatomic mapping of the proximal to distal activation in the CS. This image displays the ablation lesions and roof portion of the circuit. Blue lesion denotes the termination site. *Point at which the ERP of the tissue is reached and the tissue becomes refractory. 3D: three-dimensional; CS: coronary sinus; RSPV: right superior pulmonary vein; LAA: left atrial appendage; RIPV: right inferior pulmonary vein; LSPV: left superior pulmonary vein.

By evaluating both activation maps individually, it becomes apparent that the unusual, alternating pattern in the CS is because the effective refractory period (ERP) of the tissue in the atrium, at the 12 o’clock position of the mitral valve, was reached with tachyarrhythmia no. 1. Generally, the ERP must be between the CLs of the two different circuits. With one beat, the tachyarrhythmia travels in both a clockwise and counterclockwise pattern around the mitral valve, with both waves colliding just superior to the CS. With the next beat, however, the ERP of the tissue (see asterisk in **Figure 4**) is reached and the tissue becomes refractory. The tachycardia then travels in a clockwise-only pattern around the mitral valve, leading to a distal to proximal pattern in the CS. In this patient, the tachycardia mechanism was a macroreentrant rhythm, with the earliest activation noted in the 12 o’clock position, which then traversed the mitral valve to the floor of the LA near the right inferior PV. From there, the tachycardia traveled up the posterior wall from an inferior to superior location and then across the roof. The tachycardia subsequently moved towards the left atrial appendage and back towards the mitral valve. Unfortunately, this tachycardia was not stable and we were unable to perform pacing maneuvers such as postpacing intervals to confirm this. As both tachycardias utilized the tissue between the left atrial appendage and the mitral valve, this area was targeted for ablation. With the seventh ablation lesion, the tachycardia terminated abruptly **(Figure 5)**. With an isoproterenol infusion and rapid burst pacing from the high right atrium, the tachycardia was not inducible and the case was terminated. At the time of six months’ follow-up, the patient had remained in normal rhythm and off antiarrhythmic medications.

The differential for this arrhythmia includes dual atrial tachycardias originating from the right and left atria. A tachycardia from the right atrium would give a proximal to distal pattern in the CS while an LA tachycardia would give a distal to proximal pattern in the CS. As the tachycardia terminated with LA ablation only, this was unlikely, as a right atrial arrhythmia could not be terminated with LA ablation. Another differential is dual atrioventricular nodal physiology with an eccentric, leftward extension. A tachycardia that alternates between a septal pathway would show a proximal to distal pattern in the CS, while conduction over the leftward extension of the dual atrioventricular nodal physiology would have a distal to proximal pattern in the CS. This was also unlikely because there was no evidence of dual atrioventricular nodal physiology during electrophysiology study. Another differential includes a right atrial tachycardia with intermittent retrograde ventricular arrhythmia conduction through an accessory pathway. With one beat, the atrial tachycardia would travel anterogradely through the normal conduction system, producing a proximal to distal pattern in the CS. With the other beat, the tachycardia would continue to the LA via a left lateral pathway and produce a distal to proximal pattern in the CS. However, this was again unlikely, as there was no retrograde conduction during the electrophysiology study.

## Conclusion

An alternating pattern in the CS has been previously described but as the result of dual accessory pathways and not because of ERP within the LA.^3^ To our knowledge, this is the first case report to describe the use of a 3D mapping system to localize and successfully ablate an alternating pattern in the CS.
